# Screening for beta thalassaemia

**DOI:** 10.4103/0971-6866.64934

**Published:** 2010

**Authors:** Mary Petrou

**Affiliations:** Haemoglobinopathy Genetics Centre, University College London, Institute of Women’s Health, University College London Hospitals NHS Foundation Trust, Pathology Division 86-96 Chenies Mews London WC1E 6HX

As we approach the end of the first decade of the new millennium it seems a good time to reflect on the enormous changes that have taken place in the field of genomics. The human genome project (HGP) is an important feature in the molecular revolution, it involved an international effort to create an ordered map of the human genome and to make it available worldwide. The HGP was completed in 2003 and the detailed knowledge of the human genome will provide new avenues for advances in medicine and biotechnology. These advances will touch the lives of many but what is the fate for developing countries?

Prior to the HGP there were existing genomic advances, such was the case for one of the most common genes in the world; thalassaemia. We were unlikely to learn more about any disease than we already knew about thalassaemia. We know the gene, the mechanism of loss of function, yet we do little for the majority of the patients. We know the mutations that cause thalassaemia, there is reliable technology for carrier detection and molecular technology for prenatal diagnosis but it is little used in countries where the incidence of thalassaemia is high such as India.

The HGP invested large sums of funds to address the Ethical, Legal and Social issues (ELSI) that may arise from the project. However, one of the major ELSI issues that has not been addressed is the inability for the knowledge acquired to be translated for the benefit of developing countries and at the most basic level how to provide optimum treatment to the thalassaemia patients, how to use the existing technology to provide national prevention programmes for countries such as India.

Haemoglobin disorders constitute the most common lethal inherited disorders worldwide.[1] They are common in populations in tropical Africa, Asia and the Mediterranean region and have spread by migration throughout the world.[2,3] It is estimated that 307,900 children are born annually with a severe haemoglobin disorder and 60-70% of births occur in sub-Saharan Africa.[4] Consequently, sickle cell disease (SCD) accounts for 70% of haemoglobin disorders worldwide because of the high frequency of the gene. In Africa it is estimated that 224,200 infants are born annually with a sickle cell disorder and most die before they reach the age of five.[5,6] Thalassemia is prevalent in the Mediterranean area, the Middle East and South East Asia, and the Pacific. The carrier rates range from 2-19% in the different populations.[7] The birth prevalence of the haemoglobin disorders in countries affected by migration of populations varies according to the geographic location and the origin of the populations.[8]

Treatment of thalassaemia involves lifelong treatment. Management includes regular blood transfusions, iron chelation treatment, management of complications including osteoporosis, cardiac dysfunction, endocrine problems, hepatitis B and C infection, HIV infection. Life-expectancy for thalassaemia has improved significantly with modern medical treatment.[9–11] But it has been estimated that only 5-10% thalassaemic children born in India receive optimal treatment.[12] Without access to regular chelation treatment and medical care, the majority of children with thalassaemia major do not reach the age of 20.

In this issue Madan and colleagues[13] from the ICMR at the KEM Hospital in Mumbai and University College of Medical Sciences in Delhi, highlight a major public health burden in India. They analyse data of a two-centre study of 11,090 school children and determine the frequency of beta thalassaemia in India. Their data shows an overall frequency of 4.05%, with the population at 1.2 billion and birth rate of 23/1000, and using the Hardy-Weinberg equation estimates the affected thalassaemia births to be 11,316 per year added to the existing affected patients. This number is more than previously estimated.[14] They note that beta thalassaemia is present in the majority of castes, religious groups and population groups. They urge that suitable control measures be urgently taken in India. They state that the observed incidence in the different population groups will permit national prevention programmes for thalassaemia to be planned and carried out. They also give estimates for anaemia among these children, which should be revaluated using the normal haemoglobin levels for the Indian population rather than WHO criteria.

A national prevention programme was the aim of a project supported by the overseas development administration of the United Kingdom through the British council division in Mumbai and our centre, University College (London) Hospital between 1989-1994. Although the joint collaborative project provided some stimulus to the problem of thalassaemia in India and set up an excellent prevention centre in Mumbai,[15] a national prevention programme was not achieved. Twenty years later, statements stressing the importance of a prevention programme in India appear in the majority of thalassaemia screening and prenatal diagnosis publications from India.

Couples who are at risk for producing children with haemoglobin disorders have the option of avoiding the birth of an affected child with prenatal diagnosis. The births of affected children have been reduced in many Mediterranean countries by screening, counselling, the offer of prenatal diagnosis and selective termination of affected foetuses.[16–18] However, worldwide only a very small proportion of affected births are prevented by prenatal diagnosis.

We know that beta thalassaemia carriers are easily detected using automated cell counters and automated High Pressure Liquid Chromatography (HPLC) or similar methods. An important constraint in effective large screening, especially in countries where the prevalence of thalassaemia is high are limited resources, India is one such country. Therefore prevention of thalassaemia in the majority of these countries will depend on effective screening strategies and the use of strategies to optimise the cost-benefit ratio of mass screening. Studies have shown that the naked eye single tube red cell osmotic fragility test (NESTROFT) can be a very useful screening tool for beta thalassaemia trait[19,20] and is particularly attractive in a screening programme for India because of its low cost.

Many research papers emerge from India; the beta thalassaemia mutations present at the regional level are known[21] so the platform has been more than ready for a long time to implement a prevention programme. The technology for prenatal diagnosis is available in many parts of India, but is carried out in an ad hoc manner, the majority of women already having an affected child.[15,22–28]

In recent years both government agencies and non-government organisations (NGOs) in India have initiated programmes to deal with the problem. However, there is still no coordinated national thalassaemia control policy. A recent newspaper article in the Indian Express (21 May 2010) states that the Red Cross and the Ahmedabad Panchayat authorities will be screening married women with no existing children in the Ahmedabad district.

## When is the best time to offer screening in India?

It is often thought that affected births can be prevented if at-risk couples are identified prior to marriage, on the assumption that they will then decide to separate and each find another, non-carrier partner. Marriage can be a complex social phenomenon that involves many other family members besides the prospective couple, and marriage partners usually are selected either because of a strong personal preference, or for valid family or traditional reasons, or a mixture of all three.

If a planned marriage is broken because both partners carry thalassaemia this can cause social embarrassment or stigma to the young couple and their families, and there is a risk that the problem will recur if the new partners found are also carriers for the same disorder. For example, if population carrier frequency is 6%, the chance that one or both new partners will be a carrier is 12%. Therefore the recurrence risk for the couples is 12% (or even higher if the new potential partner is a relative). Being a carrier in India may render an individual unfit as a suitable marriage partner and testing after marriage or prenatal counselling would be more acceptable to the majority. The stigma associated with being a carrier can only be reduced significantly through greater awareness and public education perhaps by involving community leaders and people who are involved with arranging marriages.[29] Reduction of stigma will take years and years of public education.[30] India cannot afford to wait this long before a programme is established, there is the urgent need to start a prevention programme now, education will need to go hand in hand with a prevention programme.

Recently, mandatory premarital screening for thalassaemia and sickle cell has been conducted in Saudi Arabia with the objective of decreasing at-risk marriages. However, following counselling almost 90% of couples married despite being aware of their risk.[31] In Iran without prevention there would be approximately 1,200 affected children born annually, there are over 20,000 children attending treatment centres. Iran has taken on the vast task of providing national premarital screening and genetic counselling. By the end of 2001, over 2.7 million prospective couples had been screened and 10,298 at-risk couples identified and counselled. Fifty-three per cent of these couples proceeded with their marriage plans, 29% of at-risk couples separated, and the remainder were still struggling with their decision.[32] Further recent data shows that the number of couples proceeding with marriage has increased even further (A. Samavat personal communication). Therefore the majority of couples find it unacceptable to select a partner on the basis of genetic screening information and there is a high demand for prenatal diagnosis. A similar approach was tried in Cyprus much earlier, when marriages between carriers were actively discouraged. This approach proved to be unacceptable to the population and was soon abandoned.[33] Once prenatal diagnosis became possible for thalassemia, it was made available within the Cypriot health service. Soon after, confidential premarital screening was made mandatory among Greek Cypriots by the Greek Orthodox Church and among Turkish Cypriots by the civil authorities. It was then found that 98% of at-risk couples detected just prior to marriage proceed to marry. Nevertheless, the annual number of new births of children with thalassaemia major has decreased almost to zero in Cyprus, because couples use the information on genetic risk in a variety of ways to obtain a healthy family. Less than 5% of the decrease in thalassaemia major births is due to separation of engaged couples.

Another option is to test high school or university students and several programmes take place in India. Colah and colleagues[34] attempted to assess the impact of screening and counselling high school children for beta thalassaemia on a programme that was undertaken between 1984-1988 on 5682 school children. One hundred and fifty-three individuals were found to be carriers and counselling was provided to the families of 71 children; after a gap of 20 years an attempt was made to follow them up. Forty-seven of the 71 families were contactable but none of the 41 individuals who were married had revealed carrier status or had their partners tested before marriage. Eleven had their spouses tested after marriage. One couple had a thalassaaemic child. Therefore screening high school children with one-time counselling is insufficient to make an impact.

Cascade screening for beta thalassaemia may be another realistic option.[35] Ahmed and colleagues found that using this approach identified 31% of carriers within the families in Pakistan. In Sardinia more than 90% of the at-risk couples were identified by examining only 11% of the population.[36] This has been tried in India by Gorakshakar and colleagues[37] who identified 151 from 691 individuals. Therefore cascade screening appears to be one of the most cost-effective approaches to identify carriers of beta thalassaemia.

It has been shown in several programmes that antenatal screening is not the most effective strategy.[38] A study by Colah and colleagues[39] where antenatal screening was offered to women attending a hospital in Mumbai catering mainly to the lower social economic groups, found that only 15.4% of women booked in the first trimester of pregnancy. The women who were found to be carriers were counselled but only 29.5% attended for follow-up with their husbands. Two at-risk couples were identified and both opted for prenatal diagnosis. Therefore antenatal screening is not the ideal solution in India firstly because of late booking and secondly because pregnancy is not the ideal time to inform women that they may be at risk of producing children with a serious genetic disease. Nevertheless it is practised in several countries including the UK.

The data show us that a combination of screening approaches may be suitable for India. Interestingly, prevention programmes are supported by the majority of thalassemia major patients. It is possible that the continual birth of affected patients will ultimately have a detrimental effect on the treatment of the existing patients. For instance, if there was no prevention program in Cyprus there would be approximately 50 affected births per year. By 2021, a total of 70,000 units of blood will be required and 17.5% of the possible 400,000 possible donors (out of a population of 600,000) could need to donate blood at least once per year.[40] There is also the cost of iron chelation treatment including the cost of the remainder of the treatment, which will ultimately become prohibitive to the economy of Cyprus. There are also unspoken reasons why patients support prevention programme, like the financial burden, the difficulty in obtaining safe blood (infected with Hepatitis B, C, and HIV.) The pain and anxiety that families go through is insurmountable and the Quality of Life for most patients is dismal. Therefore it is not surprising that prevention programmes are supported by the majority of thalassaemia major patients. For India, the introduction of a prevention programme will give more hope to the thousands of existing patients.

From India there is a wealth of information on thalassaemia, but the magnitude of the problem is highlighted again in the paper by Madan and colleagues. Resources do not allow effective treatment for thalassaemia, what hope is there for these patients if a prevention programme is not implemented in India? Unlike many other genetic disorders, when carriers cannot be easily identified, the thalassaemias give us enormous opportunity to implement effective national screening programmes, to control affected births by community information, screening, counselling and prenatal diagnosis. Unless we all concentrate our efforts to implement a screening programme in India and indeed in India’s neighbours Pakistan and Bangladesh, so that at-risk couples can be offered a choice of preventing the birth of affected children, the wealth of information we have is rendered useless. The only solution for India is to implement a National Screening Programme immediately, but this can only be successful if there is political will. There appear to be many NGO efforts to deal with the problem, but these efforts have to be brought together and a policy evolved and submitted to the government for a fundable comprehensive programme.

A proportion of the 3-5% (around $1 million) of the HGP budget spent on ELSI Legal could have been spent on addressing implementation of genomic advances in the developing world for the benefit of the patients.
